# Subcapsular Sinus Macrophage Fragmentation and CD169^+^ Bleb Acquisition by Closely Associated IL-17-Committed Innate-Like Lymphocytes

**DOI:** 10.1371/journal.pone.0038258

**Published:** 2012-06-01

**Authors:** Elizabeth E. Gray, Sherree Friend, Kazuhiro Suzuki, Tri Giang Phan, Jason G. Cyster

**Affiliations:** 1 Howard Hughes Medical Institute and Department of Microbiology and Immunology, University of California San Francisco (UCSF), San Francisco, California, United States of America; 2 Amnis Corporation, Seattle, Washington, United States of America; 3 Garvan Institute of Medical Research, Sydney, New South Wales, Australia; 4 St Vincent's Clinical School, Faculty of Medicine, University of New South Wales, Sydney, Australia; Ulm University, Germany

## Abstract

Subcapsular sinus macrophages (SSMs) in lymph nodes are rapidly exposed to antigens arriving in afferent lymph and have a role in their capture and display to B cells. In tissue sections SSMs exhibit long cellular processes and express high amounts of CD169. Here, we show that many of the cells present in lymph node cell suspensions that stain for CD169 are not macrophages but lymphocytes that have acquired SSM-derived membrane blebs. The CD169 bleb^+^ lymphocytes are enriched for IL-17 committed IL-7Rα^hi^CCR6^+^ T cells and NK cells. In addition, the CD169 staining detected on small numbers of CD11c^hi^ dendritic cells is frequently associated with membrane blebs. Counter intuitively the CD169 bleb^+^ lymphocytes are mostly CD4 and CD8 negative whereas many SSMs express CD4. In situ, many IL-7Rα^hi^ cells are present at the subcapsular sinus and interfollicular regions and migrate in close association with CD169^+^ macrophages. These findings suggest SSMs undergo fragmentation during tissue preparation and release blebs that are acquired by closely associated cells. They also suggest an intimate crosstalk between SSMs and IL-17 committed innate-like lymphocytes that may help provide early protection of the lymph node against lymph-borne invaders.

## Introduction

Subcapsular sinus macrophages (SSMs) are a unique subset of lymph node macrophages that form a dense layer overlapping with the lymphatic lining that separates the lymphatic sinus and B cell follicle. In situ staining has shown that SSMs express high amounts of the sialic acid binding Ig-like lectin 1 (Siglec1 or CD169) and the integrin CD11b (Mac1), and, in contrast to their counterparts in the medulla, lack expression of the macrophage marker F4/80 [1], [2], [3], [4]. Many SSMs straddle the lymphatic lining cells at the base of the subcapsular sinus, extending a “head” into the sinus and long cellular processes (or “tails”) into the adjacent B cell follicle [1]. In contrast to the dynamic behavior of dendritic cell processes [5], [6], [7], real time imaging studies have revealed that the long cellular processes of SSMs are relatively static, potentially indicating tight adhesion to adjacent stromal cells or extracellular matrix [1], [2], [3], [4].

Due to this unique localization, SSMs are poised to rapidly encounter pathogens and antigens that reach the lymph node via the lymph. Indeed, a number of studies have revealed that SSMs have the capacity to capture a range of antigens, including viral particles, immune complexes, antigen-loaded beads, and other opsonized antigens [8]. In contrast to classical macrophages, which typically internalize and degrade antigen, SSMs are thought to be poorly phagocytic [9], [10], a property that may contribute to their capacity to function as antigen-presenting cells for B cells. Antigen captured by SSMs is displayed on macrophage “tails” that extend into the B cell follicle, where B cells can directly acquire antigen via complement or B cell receptors [1], [2], [3], [4]. SSMs have also been shown to activate iNKT cells. Subcutaneously injected α-GalCer-coated microspheres were captured by SSMs, processed, and presented via CD1d to iNKT cells [11]. In addition to these antigen presentation functions, a number of recent studies have shown that SSMs are an early site of replication for a number of viruses [12]–[13] as well as the parasite *Toxoplasma gondii* [14]. SSMs have been suggested to be unusually permissive for pathogen infection, a property that might foster local production of cytokines that protect other cell types from infection [15].

As part of a continued effort to understand the biology of SSMs, attempts to isolate and study these CD169^hi^ cells have been made by us [2] and others [3], [15], [16], [17], [18]. Here we report that many of the cells in lymph node cell suspensions that stain for CD169 are lymphocytes that have acquired SSM-derived membrane blebs and thus masquerade as CD169^hi^ SSMs during flow cytometric analysis. This acquisition does not appear to be a random process as CD169 bleb^+^ lymphocytes are enriched for IL-17 committed IL-7Rα^hi^CCR6^+^ T cells and NK cells. Moreover, in situ and real-time imaging analysis reveals that IL-7Rα^hi^ CXCR6^hi^ cells are present at the subcapsular sinus and in interfollicular regions, often migrating over the membrane processes of CD169^+^ macrophages. These observations raise the possibility of crosstalk between SSMs and innate-like lymphocyte populations that may have important roles in the early phases of lymph node immune responses.

## Results

### Detection of CD169^+^ staining on IL-7Rα^hi^CCR6^+^ lymphocytes

**Figure 1:**
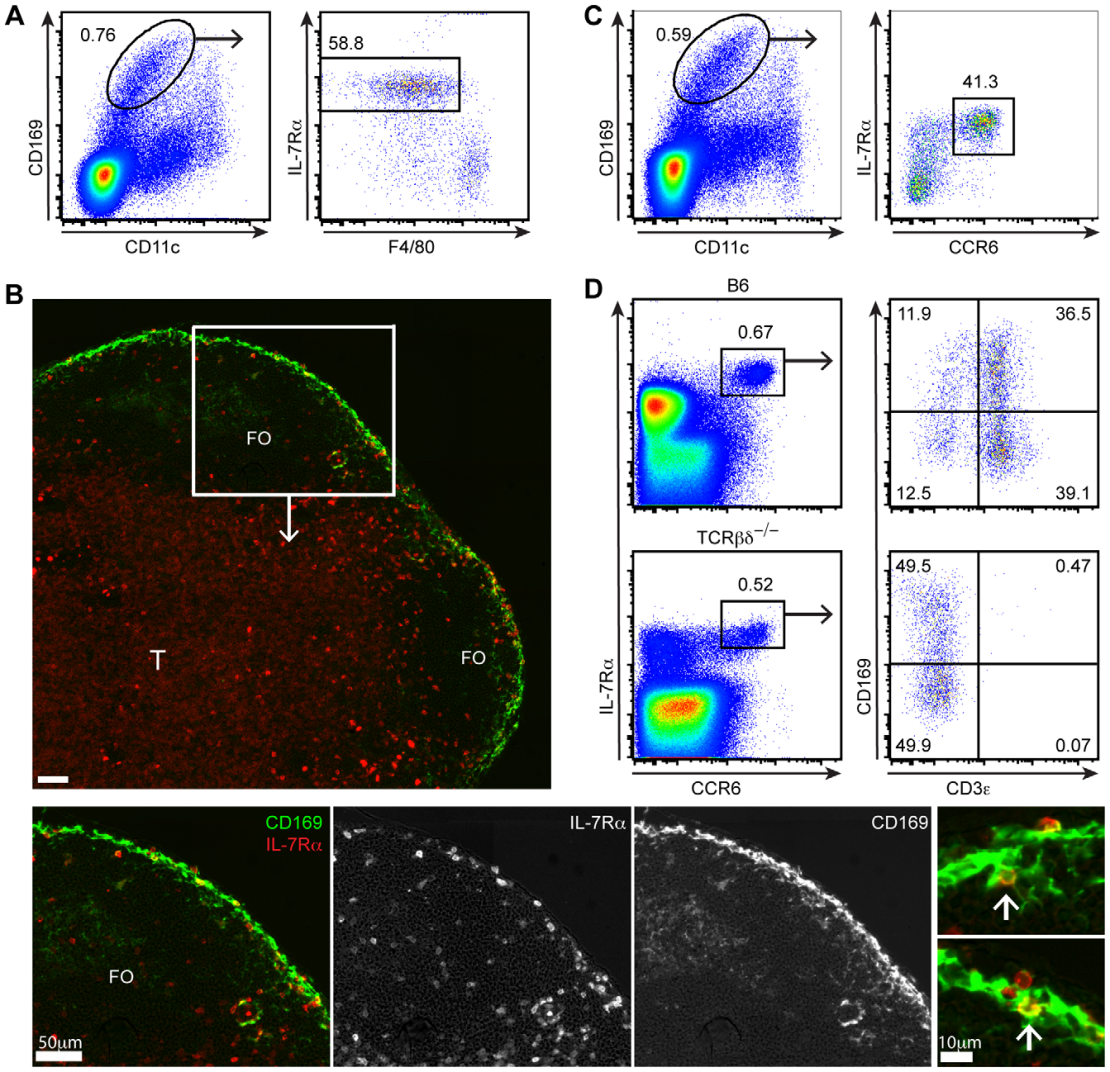
Detection of CD169^+^ IL-7Rα^hi^CCR6^+^ lymphocytes in digested lymph node tissue. (A) Flow cytometric detection of IL-7Rα expression on CD169^+^CD11c^lo^ cells from digested lymph nodes. (B) Immunofluorescence microscopy of a lymph node section stained with anti-CD169 and anti-IL-7Rα monoclonal antibodies. Enlargements (bottom, far right) show examples of IL-7Rα^+^ cells (white arrows) closely associated with CD169^+^ SSMs. FO, follicle; T, T zone. (C) Flow cytometric analysis showing CD169^+^CD11c^lo^ cells contain a population of IL-7Rα^hi^CCR6^+^ cells. All data in A–C are representative of at least three independent experiments. (D) Flow cytometric analysis of digested lymph node cells from a control and TCRβδ-deficient mouse. IL-7Rα^hi^CCR6^+^ cells gated from total cells express CD169 on a fraction of both CD3e^+^ and CD3e^−^ cells. Data are representative of at least three independent experiments (control mice) and one experiment in which a TCRβδ-deficient mouse was analyzed.

In situ staining has revealed that SSMs are CD169^hi^CD11b^+^CD11c^lo^ cells that, in contrast to the medullary macrophages, are F4/80^−^ [1], [2], [3], [4]. Previously, we identified a population of CD169^hi^CD11b^+^CD11c^lo^F4/80^−^ cells by flow cytometry that appeared to correspond to SSMs [2]. In further efforts to characterize CD169^hi^CD11c^lo^ cells by flow cytometry, we observed that many of the cells with a CD169^hi^CD11c^lo^ phenotype also expressed IL-7Rα (Figure 1A). However, subsequent analysis of IL-7Rα expression in tissue sections revealed that SSMs were IL-7Rα^−^. Instead, we observed a population of IL-7Rα^hi^ cells, with a rounded, lymphocyte-like morphology, which were present at the subcapsular sinus and interfollicular regions of the lymph node ([19] and Figure 1B and Supplemental Figure S1). These cells expressed higher amounts of IL-7Rα than the bulk population of T zone T cells and were often closely associated with the macrophages (Figure 1B). In some cases, CD169^+^ SSM “arms” appeared to be wrapped tightly around the IL-7Rα^hi^ cells (Figure 1B, far right bottom panel), although the resolution of the imaging analysis could not exclude the possibility that some IL-7Rα^hi^ cells were expressing CD169.

These results suggested that IL-7Rα^hi^ lymphocytes that stained positively for CD169 in flow cytometric analysis might have been contaminating the SSM gate. Further analysis of the IL-7Rα^hi^CD169^hi^CD11c^lo^ cells by flow cytometry revealed that the majority were CCR6^+^ (Figure 1C). When we gated on IL-7Rα^hi^CCR6^+^ cells from total lymph node cells, we observed that this gate included both CD3ε^+^ T lymphocytes and CD3ε^−^ non-T cells. Moreover, there was a “smear” of CD169 staining spanning almost a 2-log range on a fraction of both the IL-7Rα^hi^CCR6^+^ CD3ε^+^ T lymphocytes and CD3ε^−^ non-T cells (Figure 1D, top panels). CD3ε^+^IL-7Rα^hi^CCR6^+^ cells were absent in a TCRβδ-deficient mouse, confirming that a subset of the IL-7Rα^hi^CCR6^+^ cells were T lymphocytes (Figure 1D).

**Figure 2:**
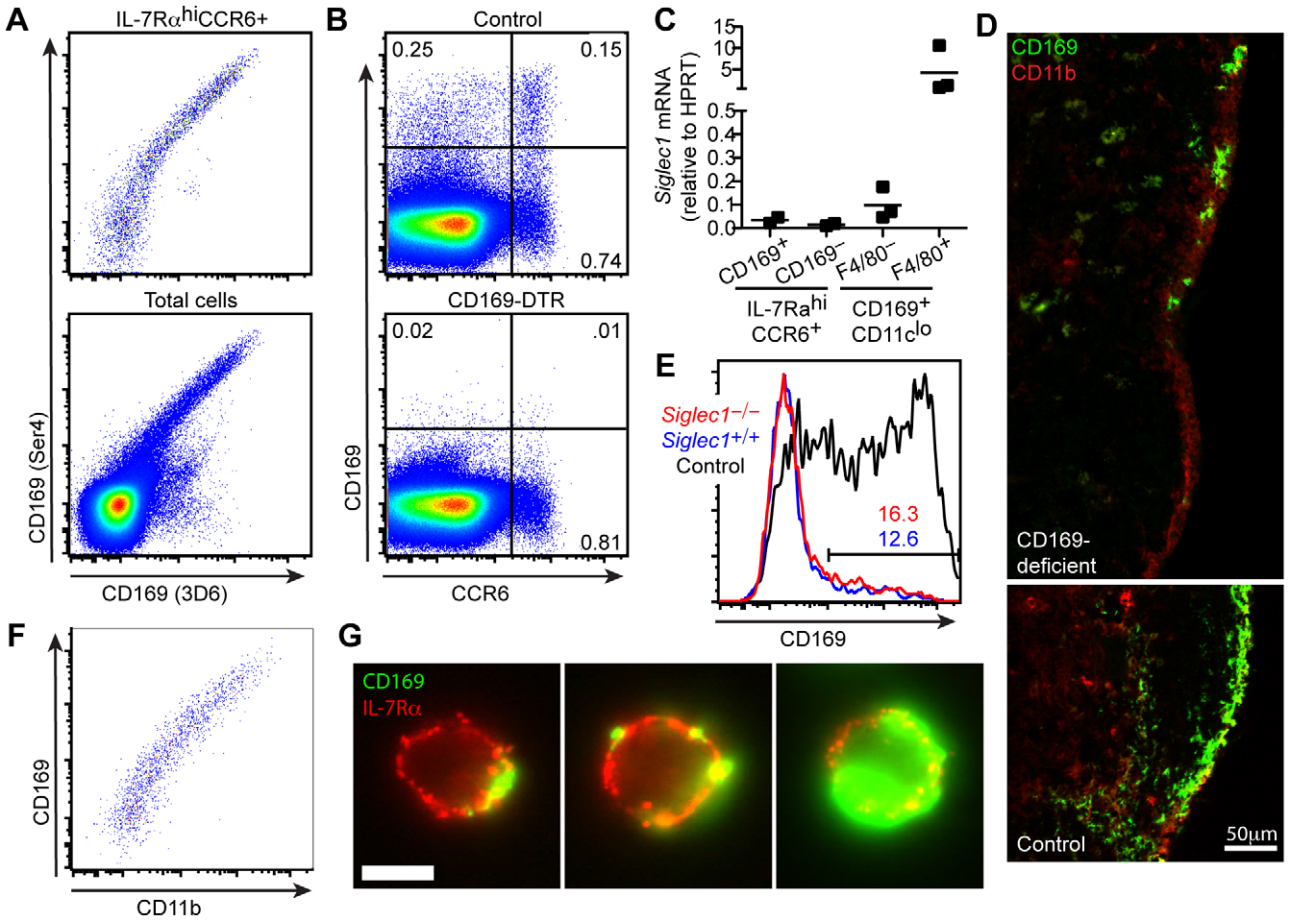
IL-7Rα^hi^CCR6^+^ lymphocytes acquire CD169^+^ SSM-derived membrane blebs. (A) Flow cytometric detection of CD169 on digested lymph node cells stained with two anti-CD169 monoclonal antibodies, Ser4 and 3D6. The top panel is pre-gated on IL-7Rα^hi^CCR6^+^ cells; the bottom panel shows total cells. Data are representative of three independent experiments. (B) Expression of CD169 on total cells from digested lymph nodes from CD169-DTR mice treated with saline or DT 3 or 4 days prior to analysis. Data are representative of two independent experiments. (C) *Siglec1* mRNA quantification by RT-PCR in sorted CD169^+^ and CD169^–^ IL-7Rα^hi^CCR6^+^ cells, CD169^+^CD11c^lo^F4/80^–^ cells, and CD169^+^CD11c^lo^F4/80^+^ cells. Data are plotted relative to HPRT. (D) Immunofluorescence microscopy of lymph nodes stained with anti-CD169 and anti-CD11b monoclonal antibodies from a *Siglec1^–/–^* bone marrow chimeric mouse (top panel) or control non-chimeric mouse (bottom). Scale bar = 50 µm. Data are representative of one experiment. (E) Flow cytometric detection of CD169 staining on CD45.1^+^ *Siglec1^+/+^* radiation-resistant (blue) compared to donor bone-marrow-derived CD45.2^+^ *Siglec1^–/–^* (red) IL-7Rα^hi^CCR6^+^ cells. CD169^+^ staining on IL-7Rα^hi^CCR6^+^ cells from a non-chimeric control animal are represented in black. Data are representative of two experiments. (F) Flow cytometric detection of CD169 and CD11b on IL-7Rα^hi^CCR6^+^ cells from digested lymph nodes. Data are representative of two experiments. (G) CD169^+^IL-7Rα^hi^CCR6^+^B220^−^ cells from digested lymph nodes were sorted and fixed to a slide for immunofluorescence microscopy. Data are representative of one experiment.

To exclude the possibility that the CD169 antibody Ser4 was cross-reacting with a non-CD169 epitope on IL-7Rα^hi^CCR6^+^ cells, we stained lymph node cell suspensions with 3D6, an antibody that recognizes a distinct epitope on CD169 [20]. IL-7Rα^hi^CCR6^+^ lymphocytes that bound Ser4 also stained with 3D6 (Figure 2A), suggesting that CD169 was present on the lymphocytes. As a further test that these cells were specifically staining for CD169, we utilized CD169-DTR mice, in which the diptheria toxin receptor (DTR) is knocked into the *Siglec1* locus. Intraperitoneal administration of diphtheria toxin (DT) causes ablation of CD169-expressing cells, including the SSMs in these mice [16], [21]. Following DT treatment, there was a loss of CD169^+^ cells by flow cytometry, including CCR6^+^CD169^+^ cells (Figure 2B), indicating that CD169 staining on IL-7Rα^hi^CCR6^+^ lymphocytes is specific.

However, when we measured CD169 transcripts on sorted CD169^+^ and CD169^−^ IL-7Rα^hi^CCR6^+^ lymphocytes, we detected low levels of *Siglec1* mRNA in both the CD169^+^ and CD169^−^ fraction (Figure 2C). In contrast, *Siglec1* mRNA was abundant in sorted CD169^+^CD11c^lo^F4/80^+^ cells (i.e. cells that stain positive for medullary sinus macrophage markers) (Figure 2C). These data do not exclude the possibility that IL-7Rα^hi^CCR6^+^ cells do intrinsically express low levels of *Siglec1* mRNA. However given the low abundance of mRNA detected in both CD169^+^ and CD169^−^ IL-7Rα^hi^CCR6^+^ lymphocytes, despite more than a 10-fold difference in CD169 staining by flow cytometry, we wondered whether these cells were acquiring CD169 in trans from other cells.

### IL-7Rα^hi^CCR6^+^ lymphocytes acquire CD169^+^ SSM-derived membrane blebs

To test whether IL-7Rα^hi^CCR6^+^ cells were acquiring CD169 in trans from other cells, we analyzed chimeras in which irradiated *Siglec1^+/+^* (CD169-sufficient) mice were reconstituted with congenically-distinct *Siglec1^−/−^* (CD169-deficient) bone marrow. Analysis of tissue sections established that the majority of CD169^hi^ SSMs were replaced by donor-derived *Siglec1^−/−^* cells in these animals; however, a small fraction of radiation-resistant CD169^+^ macrophages remained (Figure 2D), consistent with earlier findings [2]. We also found that a fraction of the IL-7Rα^hi^CCR6^+^ lymphocytes were radiation resistant such that about 1/3 of the cells with this phenotype in the chimeras were host-derived CD45.2^+^ cells (not shown). Analysis of CD169 expression by IL-7Rα^hi^CCR6^+^ lymphocytes isolated from the chimeric mice revealed reduced CD169 staining compared to IL-7Rα^hi^CCR6^+^ lymphocytes from non-chimeric *Siglec1^+/+^* mice (Figure 2E). However, CD169 staining was similar on CD45.2^+^ *Siglec1^−/−^* donor BM-derived IL-7Rα^hi^CCR6^+^ lymphocytes and CD45.1^+^ *Siglec1^+/+^* radiation-resistant host cells (Figure 2E). Since the *Siglec1^−/−^* cells cannot express CD169 endogenously, they must have acquired the CD169 in trans, most likely from the few wild-type SSMs remaining in these chimeric mice. Analysis of mixed bone marrow chimeras that were reconstituted with ∼5% CD45.2^+^ *Siglec1^−/−^* cells and ∼95% CD45.1^+^ *Siglec1^+/+^* cells also revealed no difference in CD169 staining on *Siglec1^−/−^* and *Siglec1^+/+^* IL-7Rα^hi^CCR6^+^ cells (Supplemental Figure S2A), confirming that these lymphocytes were acquiring CD169 in trans.

These combined data led us to hypothesize that IL-7Rα^hi^CCR6^+^ lymphocytes acquire CD169^+^ macrophage-derived membrane fragments or “blebs”. Consistent with this possibility, we observed that CD169 staining intensity on IL-7Rα^hi^ lymphocytes correlated with other markers expressed by SSMs based on in situ staining, such as CD11b (Figure 2F). To explore the possibility that these cells acquire CD169^+^ blebs, CD169^+^ IL-7Rα^hi^CCR6^+^ lymphocytes were sorted and fixed to slides to visualize CD169 staining. CD169-staining membrane blebs were observed attached to the surface of IL-7Rα^hi^CCR6^+^ lymphocytes (Figure 2G). Consistent with the range in CD169 staining intensity observed by FACS, we noted significant variation in the number and size of blebs attached to each cell. In some cases the amount of CD169 surface staining was extensive. However, even in many of these cases, the marker did not appear uniformly distributed across the plasma membrane, suggesting that the staining was associated with a macrophage-derived membrane process that encompassed a large part of the lymphocyte surface (Figure 2G, far right panel).

**Figure 3:**
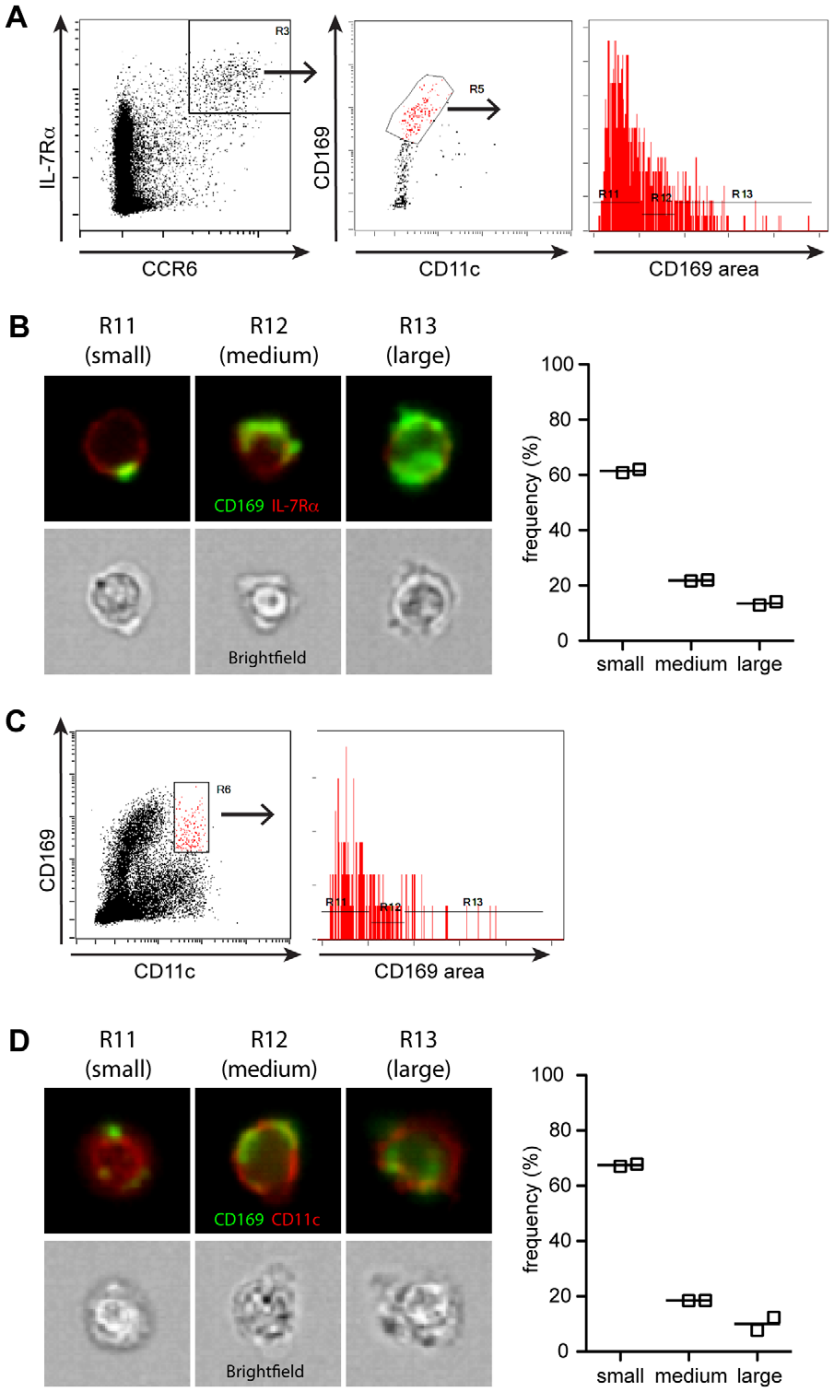
Analysis of CD169 distribution on IL-7Rα^hi^CCR6^+^ lymphocytes and CD11c^hi^ dendritic cells using ImageStreamX imaging flow cytometry. (A) Gating scheme to identify CD169^+^IL-7Rα^hi^CCR6^+^ cells analyzed on an ImageStreamX imaging flow cytometer (Amnis Corp). The area of CD169 staining on images of the gated cells was quantified using a Threshold mask on the upper 60% of the pixel intensities (right panel). (B) Representative images of cells with small, medium, and large areas of CD169 area based on the histogram in (A). Channels for CD169 (green), IL-17Rα (red), and brightfield are shown. Graph on right shows the frequency of cells falling in each gate (n = 2 mice, lines indicate means). (C) Gating scheme to identify CD169^+^CD11c^+^ cells analyzed on an ImageStreamX imaging flow cytometer. The area of CD169 staining on images of the gated cells was quantified as described in (A). (D) Representative images of cells with small, medium, and large areas of CD169 area based on the histogram in (C). Channels for CD169 (green) IL-17Rα (red), and brightfield are shown. Graph on right shows the frequency of cells falling in each gate (n = 2 mice, lines indicate means). Data are representative of one experiment with two mice. In a second experiment with two mice, CD169^+^ blebs were visualized on CD169^+^CCR6^+^TCRγδ^+^ cells.

To assess CD169^+^ bleb acquisition by IL-7Rα^hi^CCR6^+^ lymphocytes more quantitatively, experiments were carried out using ImageStreamX imaging flow cytometry (Amnis Corp). Cells with the CD169^+^ IL-7Rα^hi^CCR6^+^ marker profile were gated and CD169 surface distribution was analyzed. Similar to our observations with sorted cells, we observed CD169^+^ “blebs” of various sizes on the surface of the cells (Figure 3A, B). To quantify the fraction of cells with small compared to large CD169^+^ “blebs”, we calculated the area of CD169 staining on the gated cells and separated the images into small, medium, and large area gates (Figure 3A, B). Most CD169^+^ IL-7Rα^hi^CCR6^+^ cells had a small area of CD169 staining, while approximately 15% had a large area of staining (Figure 3B). CD169^+^ IL-7Rα^hi^CCR6^+^ cells in the small area gate (R11) tended to have small, punctate CD169 staining, while those in the large area gate (R13) tended to have CD169 staining covering the majority of the cell (Figure 3B). Whether these rare cells with a large area of CD169 staining have acquired very large CD169^+^ blebs or actually express CD169 will require future study.

High CD169 staining was recently reported on a subset of CD11c^hi^ tumor-antigen-presenting lymph node cells [16]. Given the above findings, we wondered whether CD169^hi^CD11c^hi^ cells all express CD169, or whether some might be CD11c^hi^ dendritic cells that have acquired CD169^+^ blebs. Consistent with the latter possibility, analysis of mixed bone marrow chimeras revealed no difference in CD169 staining on *Siglec1^−/−^* and *Siglec1^+/+^* CD11c^hi^ cells (Supplemental Figure S2B). Thus, like IL-7Rα^hi^CCR6^+^ lymphocytes, CD11c^hi^ cells can acquire CD169 in trans. We then asked whether we could visualize CD169^+^ blebs on CD11c^hi^ cells using imaging flow cytometry. Indeed, analysis of gated CD169^+^CD11c^+^ cells showed bleb-like CD169 staining on many of these cells (Figure 3D). Quantification of the CD169 staining area revealed that the majority of CD169^+^CD11c^hi^ cells had a small area of CD169 staining, corresponding to CD11c^hi^ cells with small blebs of CD169 staining (Figure 3C, D). Less than 15% of CD169^+^CD11c^hi^ cells had a large area of CD169 staining, which sometimes appeared uniform and covered much of the cell (Figure 3C, D). These cells may correspond to rare CD11c^hi^ cells that express CD169 or that have acquired large CD169^+^ blebs. Together, these data suggest that the majority of CD169^+^CD11c^hi^ cells are dendritic cells that have acquired CD169+ blebs.

### CD169 bleb^+^ cells are enriched for IL-7Rα^hi^CCR6^+^ cells and NK cells

**Figure 4:**
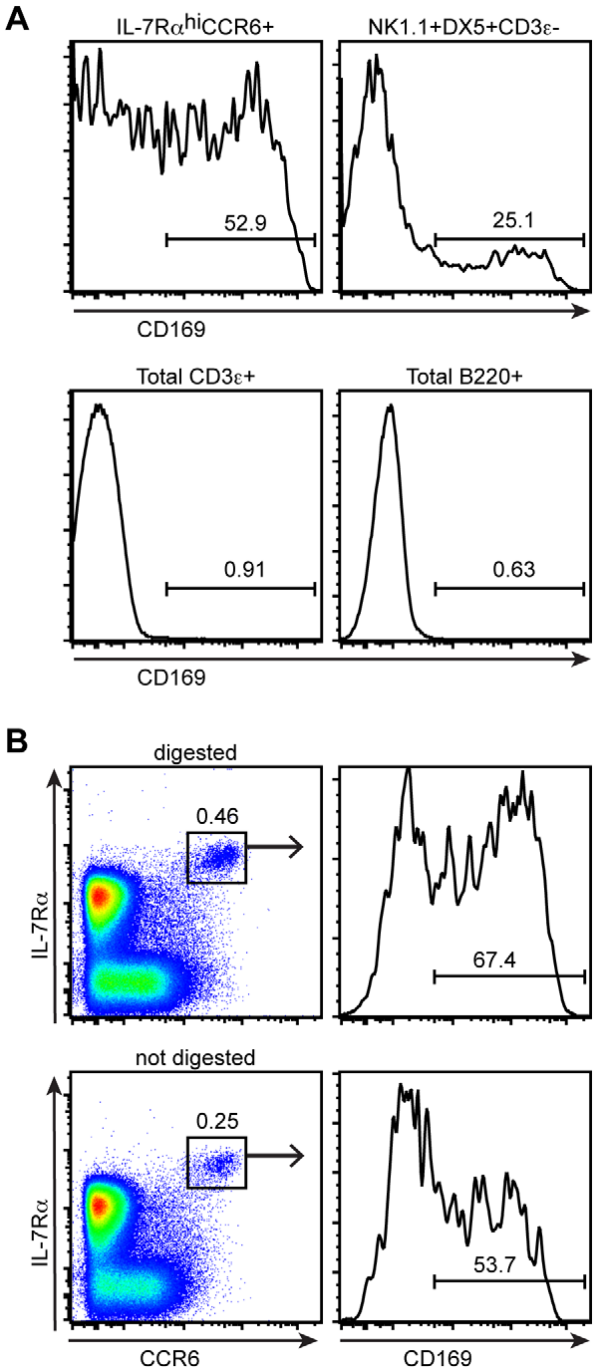
CD169 bleb^+^ cells are enriched for IL-7Rα^hi^CCR6^+^ cells and NK cells. (A) Flow cytometric detection of CD169 on digested lymph node cells gated on IL-7Rα^hi^CCR6^+^ cells, NK1.1^+^DX5^+^CD3ε^−^ cells, CD3e^+^ cells, and B220^+^ cells. (B) Flow cytometric detection of CD169 on IL-7Rα^hi^CCR6^+^ cells from digested (top panel) or non-digested (bottom panel) lymph nodes. All data are representative of at least two independent experiments.

These results suggested that IL-7Rα^hi^CCR6^+^ cells do not express CD169, but rather that they acquire CD169^+^ macrophage-derived blebs. Bleb acquisition does not appear to be a random process, as CD169^+^ staining was enriched on IL-7Rα^hi^CCR6^+^ lymphocytes compared to total T and B cells (Figure 4A). Furthermore, we observed enrichment for CD169 staining on NK1.1^+^DX5^+^CD3e^−^ NK cells, suggesting that NK cells also acquire blebs from CD169^hi^ macrophages (Figure 4A). This may suggest that these cell types have a unique capacity to capture blebs that are shed by the SSMs either in vivo or during tissue preparation. Alternatively, given that IL-7Rα^hi^ cells are located near CD169^+^ SSMs in situ (Figure 1B and Supplemental Figure S1), bleb acquisition may be dependent on localization adjacent to SSMs. Notably, tissue digestion was not required for CD169^+^ bleb acquisition as the fraction of IL-7Rα^hi^CCR6^+^ lymphocytes that were CD169^+^ by flow cytometry was similar in digested compared to undigested cell suspensions (Figure 4B). The total number of IL-7Rα^hi^CCR6^+^ lymphocytes was greater in digested samples, suggesting that while digestion improved the recovery of these lymphocytes, it was not required for bleb acquisition per se.

### IL-7Rα^hi^CCR6^+^ lymphocytes are IL-17 committed cells that interact with CD169^+^ macrophages

**Figure 5:**
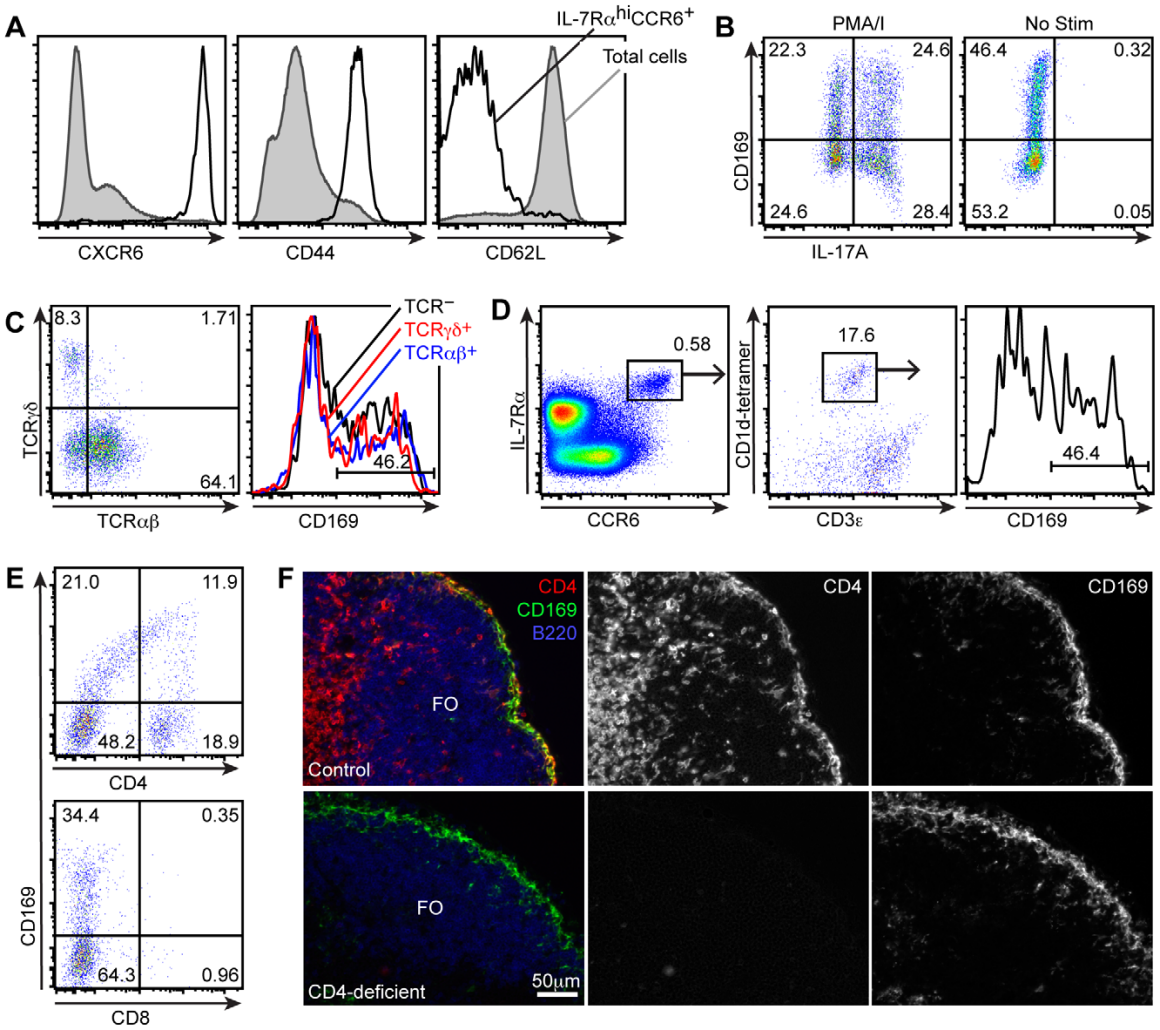
IL-7Rα^hi^CCR6^+^ lymphocytes are IL-17 producing cells. (A) CD44, CD62L and CXCR6 expression on digested lymph node cells from control or *Cxcr6^GFP/+^* mice, gated on IL-7Rα^hi^CCR6^+^ cells. (B) IL-17A staining of digested lymph node cells, stimulated with PMA/I for 2 h, gated on gated on IL-7Rα^hi^CCR6^+^ cells. (C) αβT and γδT staining on digested lymph node cells, gated on IL-7Rα^hi^CCR6^+^ cells; CD169 staining of αβT^+^, γδT^+^ and TCR^−^ IL-7Rα^hi^CCR6^+^ cells gated as indicated in the left panel. (D) Flow cytometric detection of IL-7Rα^hi^CCR6^+^ cells that bind CD1d-tetramers; far right panel shows CD169 staining on CD1d-tetramer^+^ IL-7Rα^hi^CCR6^+^ cells. (E) CD169, CD4, and CD8 staining on digested lymph node cells, gated on IL-7Rα^hi^CCR6^+^ cells. (F) Immunofluorescence microscopy of a lymph node from a wild-type or a CD4-deficient mouse, stained with anti-CD169 and anti-CD4 monoclonal antibodies. FO, follicle; T, T zone. Scale bar = 50 µm. All data are representative of at least two independent experiments.

IL-7Rα^hi^CCR6^+^ cells express high levels of CXCR6 ([19] and Figure 5A), a chemokine receptor enriched on effector and memory T cells [22]. Consistent with an effector phenotype, flow cytometric analysis revealed that IL-7Rα^hi^CCR6^+^ cells were CD44^hi^ and CD62L^lo^ (Figure 5A). Based on their expression of CCR6, a marker of IL-17 committed cells [23], we hypothesized that these cells were programmed to make IL-17A. Indeed, the IL-7Rα^hi^CCR6^+^ lymphocytes rapidly produced IL-17A following phorbol ester plus ionomycin stimulation ex vivo, although there was no difference in the capacity of CD169 bleb^+^ cells to make IL-17A compared to the bleb^−^ fraction (Figure 5B). The ability of the IL-7Rα^hi^CCR6^+^ lymphocytes to rapidly produce cytokines ex vivo in addition to their effector phenotype suggested that these cells correspond to the IL-17A committed cells recently described by several groups [24].

The IL-7Rα^hi^CCR6^+^ gate included γδT, αβT, and non-T cells (Figure 5C) all of which showed a smear of CD169 staining. The IL-7Rα^hi^CCR6^+^ γδT cells correspond to innate IL-17 producing γδT that have been described in the dermis and peripheral lymph nodes [19], [25], [26]. We observed that approximately 15–20% of IL-7Rα^hi^CCR6^+^ cells were CD1d-tetramer^+^ CD3ε^int^ iNKT cells, which showed a smear of CD169 staining (Figure 5D). These CCR6^+^ iNKT cells likely correspond to the IL-17 producing iNKT cells recently described in skin and peripheral lymph nodes [27].

Analysis of CD4 expression by the IL-7Rα^hi^CCR6^+^ T cells showed a smear of CD4 signal, much of which correlated with CD169 staining (Figure 5E, top panel, upper quadrants) as well as a population of T cells with a conventional CD4^hi^CD169^−^ phenotype (Figure 5E, top panel, lower right quadrant). In contrast, the IL-7Rα^hi^CCR6^+^ T cells were CD8^−^ (Figure 5E, bottom panel). The smear of CD4 staining suggested, counter intuitively, that the SSMs express CD4 and that the CD4^−^ IL-7Rα^hi^CCR6^+^ T cells were acquiring CD4^+^CD169^+^ blebs from the macrophages. Indeed, analysis of CD4 expression in situ revealed CD4 staining on the majority of CD169^+^ SSMs in control but not CD4-deficient mice (Figure 5F).

Finally, we took advantage of the high expression of CXCR6 by IL-7Rα^hi^CCR6^+^ cells to gain a more precise assessment of their localization in lymph node tissue sections than can be achieved by IL-7Rα-staining alone. Approximately 60–80% of IL-7Rα^hi^CXCR6^hi^ lymphocytes identified by flow cytometry are CCR6^+^ [19] (and data not shown), suggesting that the majority of IL-7Rα^hi^CXCR6^hi^ cells on sections correspond to IL-7Rα^hi^CCR6^+^ IL-17-committed lymphocytes. Using *Cxcr6^GFP/+^* reporter mice [22], we found that the IL-7Rα^hi^CXCR6^hi^ cells were abundant in subcapsular and interfollicular regions, often adjacent to CD169^+^ macrophages (Supplementary Figure S3). Moreover, two photon laser scanning microscopy of intact lymph nodes revealed CXCR6^hi^ (GFP^hi^) cells migrating in close association with CD169+ macrophages in subcapsular sinus and interfollicular regions (Supplementary Movies S1 and S2).

## Discussion

We report here that many of the cells in lymph node cell suspensions that stain positively for SSM-markers are not macrophages, but rather IL-7Rα^hi^CCR6^+^ lymphocytes and NK cells that have acquired CD169^+^ SSM-derived membrane blebs. This conclusion is established by: (i) the incomplete concordance between surface marker expression on CD169^+^ cells detected in tissue sections and by flow cytometry; (ii) the low abundance of CD169 transcripts in CD169^+^ IL-7Rα^hi^CCR6^+^ cells by quantitative PCR as well as in microarray analysis of FACS sorted CD169^+^ CD11b^+^CD11c^lo^F4/80^−^ cells [2], [28]; (iii) the positive CD169 staining on *Siglec1^−/−^* IL-7Rα^hi^CCR6^+^ cells in *Siglec1^+/+^* hosts, and; (iv) the visualization of CD169^+^ blebs on the surface of sorted cells and cells examined using ImageStreamX flow cytometry. The tendency of IL-7Rα^hi^CCR6^+^ lymph node cells to acquire CD169^+^ macrophage-derived blebs suggests there may be a propensity for these two cell types to interact in vivo. Future studies should explore whether a specific interaction between IL-7Rα^hi^CCR6^+^ lymphocytes and CD169^+^ macrophages occurs in vivo, and, if so, define the functional consequences.

An important question that arises from these observations is whether bleb acquisition by IL-7Rα^hi^CCR6^+^ lymphocytes occurs in vivo or during cell preparation. Arguing against acquisition being a prominent process in vivo, during real-time imaging studies of intact lymph nodes in mice intravitally labeled with CD169 antibodies, we have so far not observed CD169 marker acquisition by migrating lymphocytes (Supplementary Movies S1 and S2, and data not shown). Therefore, we favor the view that bleb acquisition predominantly occurs during tissue preparation. The finding that CD169 acquisition by IL-7Rα^hi^CCR6^+^ cells was similar whether the lymph nodes were gently teased apart and enzyme digested or simply mechanically separated suggests that SSMs may be highly prone to fragmentation, perhaps due to strong attachments to the surrounding cells or extracellular matrix. Alternatively, the cells may be programmed to undergoing blebbing during apoptosis. In this regard it is notable that dying germinal center B cells in intact lymph nodes can release blebs that are acquired by neighboring lymphocytes [29] and active ROCK-dependent membrane blebbing during apoptosis has been reported in a number of in vitro studies [30]. In addition, one physiological mechanism of axon pruning involves active membrane blebbing [31]. Whatever the mechanism for the SSM blebbing, the present findings highlight the challenges associated with isolating pure populations of SSMs, challenges that likely extend to other cell populations with long membrane processes, such as the closely related CD169^+^ marginal metallophillic macrophages (MMMs) in the spleen and the non-hematopoietic follicular dendritic cells in all secondary lymphoid tissues. MMM isolation and analysis by flow cytometry has been reported in a number of studies but few of these studies have examined the isolated cells by microscopy. In a report where the low density (macrophage-enriched) fraction of cells from digested spleen and lymph nodes was examined by microscopy, less than 0.1% of the cells stained for markers corresponding to MMM and SSM; these cells were noted to have a granular cytoplasm and in some cases appeared tightly associated with lymphocyte-sized cells [32].

Given that CD169 bleb^+^ cells are enriched for IL-7Rα^hi^CCR6^+^ cells and NK cells, in future efforts to isolate SSMs it will be important to include IL-7Rα, CCR6, and NK1.1 in addition to CD3ε and B220 in a lineage ‘dump’ gate, while keeping in mind that SSMs are CD4^+^. In our initial efforts to perform such an analysis we have found that there are very few lineage ‘dump’ negative cells (unpubl. obs.). However, it must also be considered likely that SSMs themselves will be strongly associated with IL-7Rα^hi^CCR6^+^ cells, causing them to be lost during doublet or ‘dump’ gating. In future studies, it will be important to perform microscopy on CD169^hi^ cells of large size, including cells that might be classified as doublets on flow cytometry, to more definitively test for the amount of SSM recovery that can be achieved with current tissue digestion procedures. Macrophage reporter mice, such as the MacGreen mice [33] or Lysozyme M-cre [34] x Rosa-stop^flox^YFP mice may be of utility in identifying intact SSMs, given that the cytoplasmic reporter molecules may be restricted to intact macrophages and absent from macrophage-derived blebs. Until improved isolation and cell tracing procedures are developed it will be important to confirm any unique properties of the cells suggested based on gene expression analysis of sorted cells [2], [15], [17] through assessments of gene or protein expression by the cells in situ or after isolation from snap-frozen tissue by laser capture microscopy [35].

Our finding that mouse SSMs express CD4 is consistent with an earlier study in rats showing CD4 expression by these cells [36]. Most other mouse macrophage populations, including peritoneal macrophages, Kupffer cells, and red pulp macrophages do not express CD4, in contrast to rats as well as humans, in which CD4 expression by monocytes and macrophages is more widespread [37]. Thus, CD4 expression may be a special feature of these lymph node macrophages. CD4 expression by human lymph node sinus macrophages, which may correspond to mouse subcapsular sinus macrophages, has been reported [38]. SSMs are thought to be uniquely permissive for viral replication [12], [15], [35] raising the possibility that expression of the HIV-coreceptor CD4 by SSMs may play an important role during HIV infection and perhaps also in the capture and display of HIV-1 virions for recognition by B cells.

We describe a population of IL-7Rα^hi^CCR6^+^CXCR6^hi^ lymphocytes that are abundant at the subcapsular sinus and in interfollicular regions. This is a diverse population of cells, including γδT, αβT, and non-T cells, all of which rapidly produce IL-17A when stimulated with PMA and ionomycin ex vivo. The IL-7Rα^hi^CCR6^+^ TCRγδ^+^ cells correspond to innate IL-17 producing γδT that have been described in the dermis and peripheral lymph nodes [19], [25], [26]. 15–20% of IL-7Rα^hi^CCR6^+^ cells were iNKT cells, which likely correspond to the IL-17 producing iNKT cells recently described in skin and peripheral lymph nodes [27]. In another study, approximately 10% of adoptively transferred iNKT cells localized to the interfollicular or subcapsular sinus regions of the lymph node [17], consistent with the notion that at least a subset of iNKT localize near SSMs in the steady state. What types of effector cells the remaining TCRβ^+^ CD4 and CD8 double negative cells and CD3ε^−^ cells in the CD169^+^IL-7Rα^hi^CCR6^+^ gate correspond to is unclear, although they may include IL-17 producing LTi-like cells [24], [39].

The presence of IL-17 committed lymphocytes in or near the subcapsular sinus and their migration in close association with CD169^+^ macrophages raises the possibility that these cell types functionally interact. The spontaneous clustering or ‘swarming’ of CXCR6^hi^ (GFP^hi^) cells in close proximity with SSMs observed in some of our imaging experiments (Supplementary Movie S2) also provides support for crosstalk between these cells though the type(s) of stimuli that provoke this behavior are not yet defined. IL-17 plays an important role in immunity at barrier surfaces, such as the skin [24]. One might consider the subcapsular sinus lining cells as a second barrier, given the dense network of macrophages and size-exclusion properties of this site as well as the constant exposure to lymph fluid that delivers antigens to the sinus within seconds to minutes of inoculation [1], [3], [40], [41]. CD169^+^ macrophages, including cells at the subcapsular sinus and in interfollicular regions, constantly sample the lymph draining the skin for antigen and likely also for inflammatory cytokines. Thus, one intriguing possibility is that SSMs sense lymph-derived signals and activate adjacent lymphocytes by producing IL-17-promoting cytokines, such as IL-1β and IL-23 [42], [43] or upregulating presentation of their cognate ligands, such as the recently described endogenous iNKT ligand, β-GlcCer [44]. Early production of IL-17 and possibly additional cytokines may enhance pro-inflammatory cytokine production and microbicidal activity of the macrophages [45], [46] and help maintain the barrier function of the subcapsular sinus while influencing the initial induction of effector T [42] and B cells [47] within the lymph node parenchyma. Future studies should explore whether crosstalk between IL-17 committed lymphocytes and SSMs plays a role in protecting the lymph node from invading pathogens and in guiding early phases of lymph node immune responses.

## Materials and Methods

### Ethics Statement

All experiments conformed to ethical principles and guidelines approved by the UCSF Institutional Animal Care and Use Committee under protocol authorization number AN087331-01.

### Mice

C57BL/6 (CD45.2^+^), Boy/J (CD45.1^+^), *Cxcr6^gfp/+^* ([22]; 005693, B6.129P2-*Cxcr6^tm1Litt^*/J), *TCRβδ* ^−/−^ ([48]; 002122, B6.129P2-*Tcrb^tm1Mom^Tcrd^tm1Mom^*/J), and CFP-transgenic ([49]; 004218; B6.129(ICR)-Tg(CAG-ECFP)CK6Nagy/J) mice were from The Jackson Laboratory (Jax), Taconic, or National Cancer Institute. *Siglec1* ^−/−^ (CD169-deficient) mice [50] were kindly provided by Dr. Paul Crocker; *Siglec1^DTR/+^* (CD169-DTR) mice [21] were kindly provided by Dr. Masato Tanaka; *Cd4^−/−^* (CD4-deficient) mice [51] on a FvB/N background and FvB/N control mice were kindly provided by Dr. Lisa Coussens.

### Tissue preparation

Unless otherwise indicated, lymph nodes were digested as described [2]. Briefly, lymph nodes were teased apart in DMEM containing penicillin/streptomycin and HEPES buffer and digested with 67 µg/ml Liberase TM (Roche) and 20 µg/ml DNAse I (Sigma) for 20 minutes while rotating. The digestion was then quenched by the addition of 10% fetal bovine serum (FBS) and 5 µM EDTA and lymph nodes were disaggregated by mashing through 100 µm nylon sieve (BD Bioscience).

### Flow cytometry

Cells were stained [2] in “FACS buffer” (PBS with 0.1% sodium azide, 2% FBS, and 1 µM EDTA) with antibodies to TCRγδ (GL3), TCRβ (H57-597), IL-17A (eBio17B7), CD196/CCR6 (140706), IL-7Rα (A7R34 or B12-1), CD3e (clone 145-2C11), CD11c (clone HL3 or N418), CD11b (clone Mac-1), CD4 (clone GK1.5), CD8 (clone 53-6.7), NK1.1 (clone PK136), CD49b (clone DX5), CD44 (clone IM7), CD62L (clone MEL-14), F4/80 (clone Cl:A3-1), CD45.2 (clone 104) and CD45.1 (clone A20) (Biolegend, BD Biosciences, eBioscience, Cedarlane Laboratories, or Invitrogen). Biotin-conjugates were detected with streptavidin Qdot® 605 (Invitrogen). PBS-57-loaded CD1d tetramers were provided by the National Institutes of Health tetramer core facility (PBS-57 is an analogue of α-galactosylceramide). Anti-CD169 (clone Ser4) was kindly provided by Dr. Paul Crocker; Molecular Probes® Monoclonal Antibody Labeling Kits (Invitrogen) were used to directly conjugate Ser4 antibody to AlexaFluor488, AlexaFluor647, or Pacific Blue dyes. FITC-conjugated anti-CD169 ([52] clone 3D6.122) was purchased from AbD Serotec. Unless otherwise indicated, the Ser4 antibody was used to detect CD169. During analysis, singlets were gated based on peak FSC-H/FSC-W and SSC-H/SSC-W. These gates encompassed more than 90% of total events and were set sufficiently widely to include singlet events of variable size while avoiding the main doublet peak.

To detect IL-17A, cells were stimulated for 2 h with 50 ng/ml PMA (Sigma) and 1 µg/ml Ionomycin (I, EMD Biosciences) in Brefeldin A (BD Biosciences), stained for surface antigens, treated with BD Cytofix Buffer and Perm/Wash reagent (BD Biosciences), and stained with anti-IL-17A.

### Cell sorting

Digested lymph node cells were blocked with anti-CD16/32 (clone 2.4G2, UCSF hybridoma core) in 5% normal mouse serum and 5% normal rat serum in DMEM/2% FBS/5 µM EDTA and stained with anti-CD169-AlexaFluor488, anti-IL-7Rα-PE, anti-CCR6- AlexaFluor647, and anti-B220-biotin. The B220-biotin conjugate was detected with streptavidin Qdot® 605 (Invitrogen). Dead cells were excluded with DAPI and CD169^+^IL-7Rα^hi^CCR6^+^B220^−^ and CD169^−^IL-7Rα^hi^CCR6^+^B220^−^ subsets were sorted on a FACSAria with a purity of at least 88%. CD169^+^CD11c^lo^F4/80^+^ and CD169^+^CD11c^lo^F4/80^–^ cells were sorted as described [2].

RNA was extracted and *Siglec1* mRNA was quantified as previously described [2]. For imaging, a fraction of sorted cells were washed with PBS containing 0.1% BSA and 0.1% sodium azide and applied to a chambered coverslip (CultureWell, Molecular Probes). After allowing cells to settle for 20 minutes at room temperature, supernatent was carefully aspirated from the coverslips. The cells were then fixed to the coverslip for 10 minutes at room temperature with 40 µl of 4% paraformaldehyde. Coverslips were washed with PBS, stained with DAPI, and then mounted onto a glass slide. Images were acquired with a Zeiss AxioObserver Z1 inverted microscope using equivalent exposure times. Optimal exposure times were determine based on the CD169 staining intensity of the majority of the sorted cells.

### Amnis ImageStreamX imaging flow cytometry

Digested lymph node cells were blocked with anti-CD16/32 (clone 2.4G2, UCSF hybridoma core) in 5% normal mouse serum and 5% normal rat serum in “FACS buffer” (PBS with 0.1% sodium azide, 2% FBS, and 1 µM EDTA) and stained with anti-CD11c-FITC, anti-IL-7Rα-PE, anti-CCR6-AlexaFluor647, anti-CD169-Pacific Blue and anti-F4/80-biotin. The F4/80-biotin conjugate was detected with streptavidin Qdot® 605 (Invitrogen). After washing, cells were fixed for 30 minutes on ice with BD Cytofix Buffer, resuspended in FACS buffer, and analyzed on an Amnis ImageStreamX imaging flow cytometer instrument. In a second experiment cells were stained with anti-CD169-AlexaFluor488, anti-TCRγδ-PE, anti-CCR6-AlexaFluor647, and anti-TCRβ-APC-eF780, and CD169^+^ blebs were visualized on CCR6^+^TCRγδ^+^ cells.

### Immunofluorescence

5-µm or 7-µm sections were prepared from paraformaldehyde-fixed tissues, prepared as previously described [53]. In some cases, acetone-fixed sections were stained as previously described [1]. Sections were stained with the following antibodies: Ser4-AlexaFluor488 or Ser4-AlexaFluor647, anti-CD4-PE (clone GK1.5), anti-IL-7Rα-PE (clone A7R34), and anti-B220-AlexaFluor647 (clone RA3-6B2). Images were acquired on a Zeiss AxioObserver Z1 or Leica TCS SP2 confocal microscope. Images were processed using Adobe PhotoShop CS2. CD4 staining by CD169^+^ macrophages was analyzed on lymph nodes from at least three C57BL/6 mice as well as two pairs of *Cd4* ^–/–^ and control mice. CD4 staining on CD169^+^ wild-type macrophages was similar in C57BL/6 and FvB/N mice.

### CD169-deficient bone marrow chimeras

Bone marrow cells (3–6×10^6^) from *Siglec1^–/–^* mice were transferred intravenously to congenic *Siglec1^+/+^*recipients lethally irradiated with a split dose of 1100–1300 rads. In some experiments, a mixture of CD45.2+ *Siglec1^–/–^* or *Siglec1^+/+^* and CD45.1+ *Siglec1^+/+^* bone marrow cells were transferred into irradiated CD45.1+ recipients. Recipient mice were analyzed at least 8 weeks later.

### Two-photon microscopy

CFP^+^ B cells were transferred intravenously and anti-CD169-biotin/streptavidin-PE was injected s.c. to label SSMs in *Cxcr6^gfp/+^* mice 20–24 hours prior to imaging. Lymph node explants were prepared for imaging as previously described [29], [54] and imaged with a Zeiss LSM 7MP equipped with a Chameleon laser (Coherent). Fluorophores were excited at 870 nm and detected with 450–490 nm (CFP), 500–550 nm (GFP) and 570–640 nm (PE) emission filters. Images were acquired with Zen (Zeiss), and time-lapse images generated with Imaris 7.4.0 (Bitplane). Videos were processed with a Gaussian noise filter. Annotation and final compilation of videos were with After Effects 7.0 software (Adobe Systems). Video files were converted to MPEG format with AVI-MPEG Converter for Windows 1.5 (FlyDragon Software). In some early experiments (imaging data not shown), images were acquired and videos generated as previously described [55].

## Supporting Information

Figure S1 **IL-7Rα^hi^ lymphocytes are located adjacent to CD169+ macrophages.** Three additional examples of lymph node sections stained as in Figure 1B to detect CD169 (green) and IL-7Rα (red). FO, follicle; T, T zone. Scale bar = 50 µm.
(TIF)

Figure S2 **CD169 staining on** ***Siglec1^–/–^*** **(CD169-deficient) IL-7Rα^hi^CCR6^+^ lymphocytes and CD11c^hi^ dendritic cells in mixed bone marrow chimeras.** Flow cytometric detection of CD169 staining on CD45.2^+^ *Siglec1^–/–^* or *Siglec1* ^+/+^ (red) compared to CD45.1^+^ *Siglec1^+/+^* (blue) IL-7Rα^hi^CCR6^+^ cells (A) and CD11c^hi^ cells (B) in mixed bone marrow chimeric mice. Data are representative of two experiments.
(TIF)

Figure S3 **IL-7Rα^hi^CXCR6^hi^ lymphocytes at the subcapsular sinus and interfollicular regions.** Immunofluorescence microscopy of lymph node sections from *Cxcr6^GFP/+^* mice stained with anti-CD169 (blue) and anti-IL-7Rα (red) monoclonal antibodies. Two examples are shown and are representative of sections from four lymph nodes from two mice. FO, follicle; T, T zone. Scale bar = 50 µm.
(TIF)

Movie S1 **Two photon imaging of CXCR6^hi^ cells migrating in association with CD169^+^ macrophages.** Intravital TPSLM showing CXCR6^hi^ cells migrate in close association with CD169^+^ macrophages (labeled with anti-CD169-PE) at the subcapsular sinus and interfollicular regions of an explanted *Cxcr6^GFP/+^* lymph node. Movie shows 12 µm maximum intensity z projection. Time is shown as hr∶min∶sec. Data in Movies S1 and S2 are representative of one experiment in which a total of five follicles in two lymph nodes were analyzed. Similar findings were observed in a second experiment of this type, in which macrophages were labeled with PE-ICs as described [2]. FO, follicle; IF, interfollicular region; SCS, subcapsular sinus.
(MPG)

Movie S2 **Two photon imaging of CXCR6^hi^ cells migrating in association with CD169^+^ macrophages.** Intravital TPSLM showing CXCR6^hi^ cells migrate in close association with CD169^+^ macrophages (labeled with anti-CD169-PE) at the subcapsular sinus and interfollicular regions of an explanted *Cxcr6^GFP/+^* lymph node. The clustering of CXCR6^hi^ cells seen in Movie S2 was observed in 4 of 10 movies. Movie shows 12 µm maximum intensity z projection. Time is shown as hr∶min∶sec. FO, follicle; IF, interfollicular region; SCS, subcapsular sinus.
(MPG)
