# Supplementary figures and images for: Subcapsular Sinus Macrophage Fragmentation and CD169+ Bleb Acquisition by Closely Associated IL-17-Committed Innate-Like Lymphocytes
Source: PLoS One. 2012 Jun 1;7(6):e38258. doi: 10.1371/journal.pone.0038258 (PMC3365896; doi:10.1371/journal.pone.0038258)

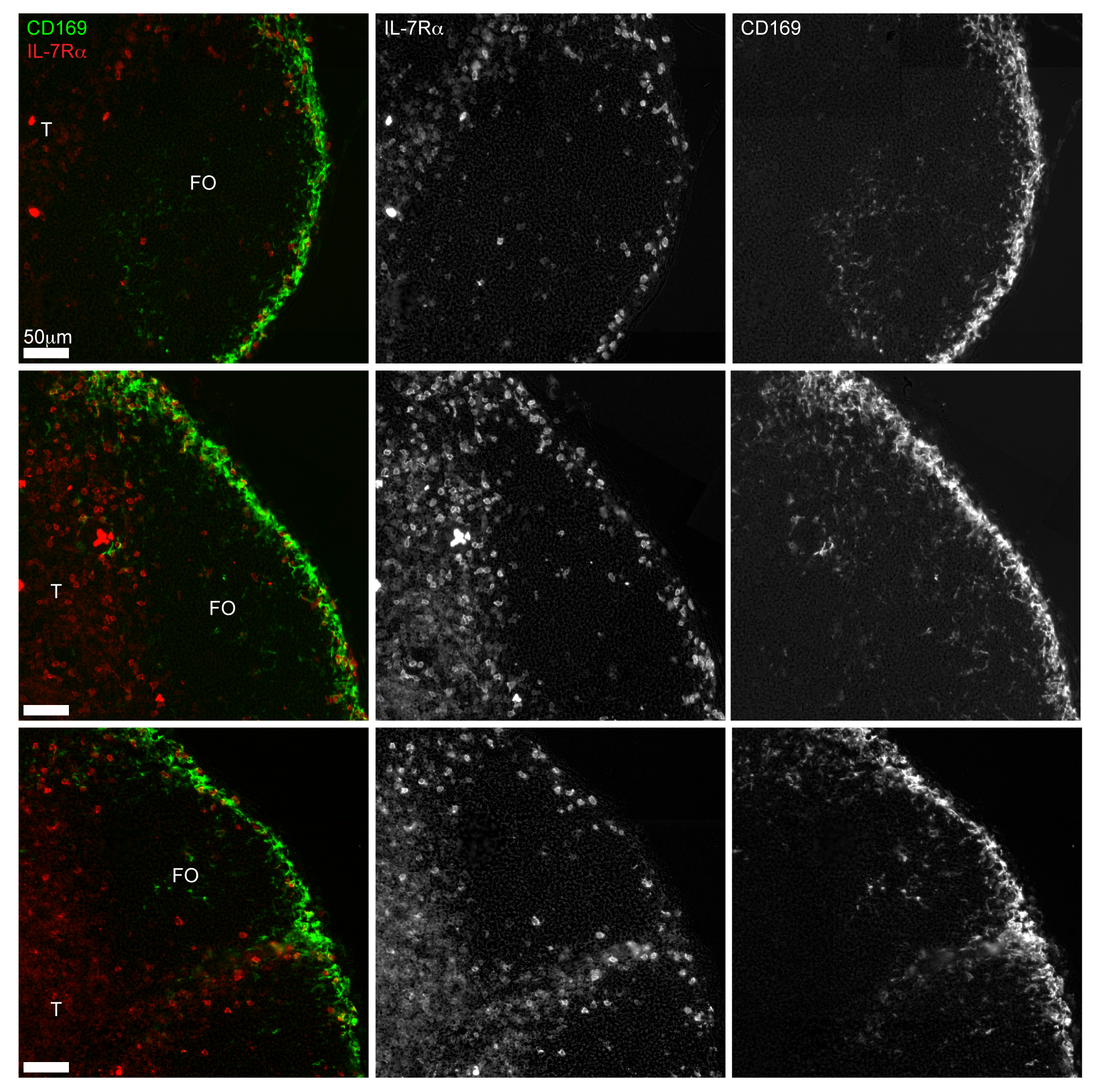

Supplement: Figure S1 — IL-7Rαhi lymphocytes are located adjacent to CD169+ macrophages. Three additional examples of lymph node sections stained as in Figure 1B to detect CD169 (green) and IL-7Rα (red). FO, follicle; T, T zone. Scale bar = 50 µm. (TIF) [file pone.0038258.s001.tif]

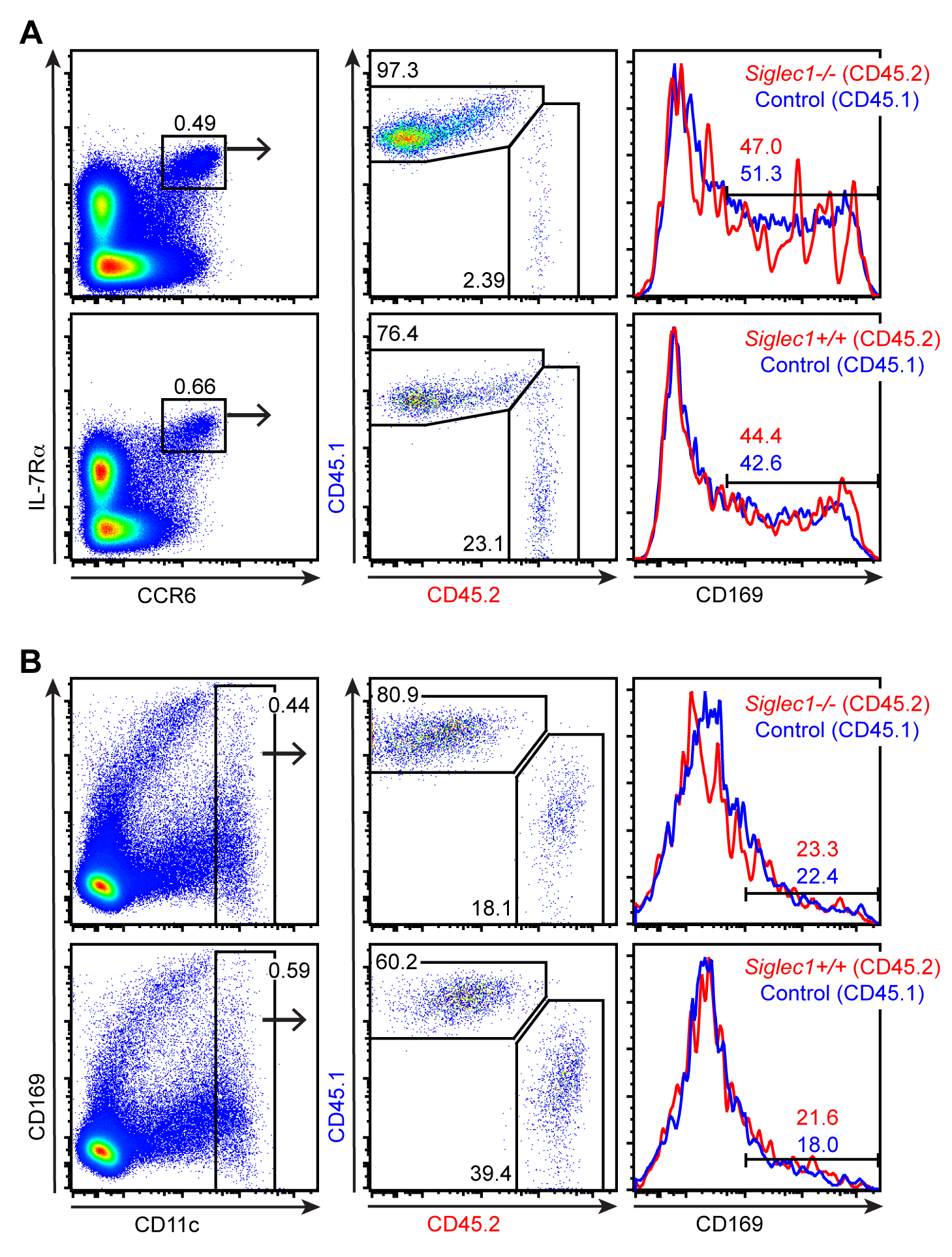

Supplement: Figure S2 — CD169 staining on Siglec1–/– (CD169-deficient) IL-7RαhiCCR6+ lymphocytes and CD11chi dendritic cells in mixed bone marrow chimeras. Flow cytometric detection of CD169 staining on CD45.2+ Siglec1–/– or Siglec1 +/+ (red) compared to CD45.1+ Siglec1+/+ (blue) IL-7RαhiCCR6+ cells (A) and CD11chi cells (B) in mixed bone marrow chimeric mice. Data are representative of two experiments. (TIF) [file pone.0038258.s002.tif]

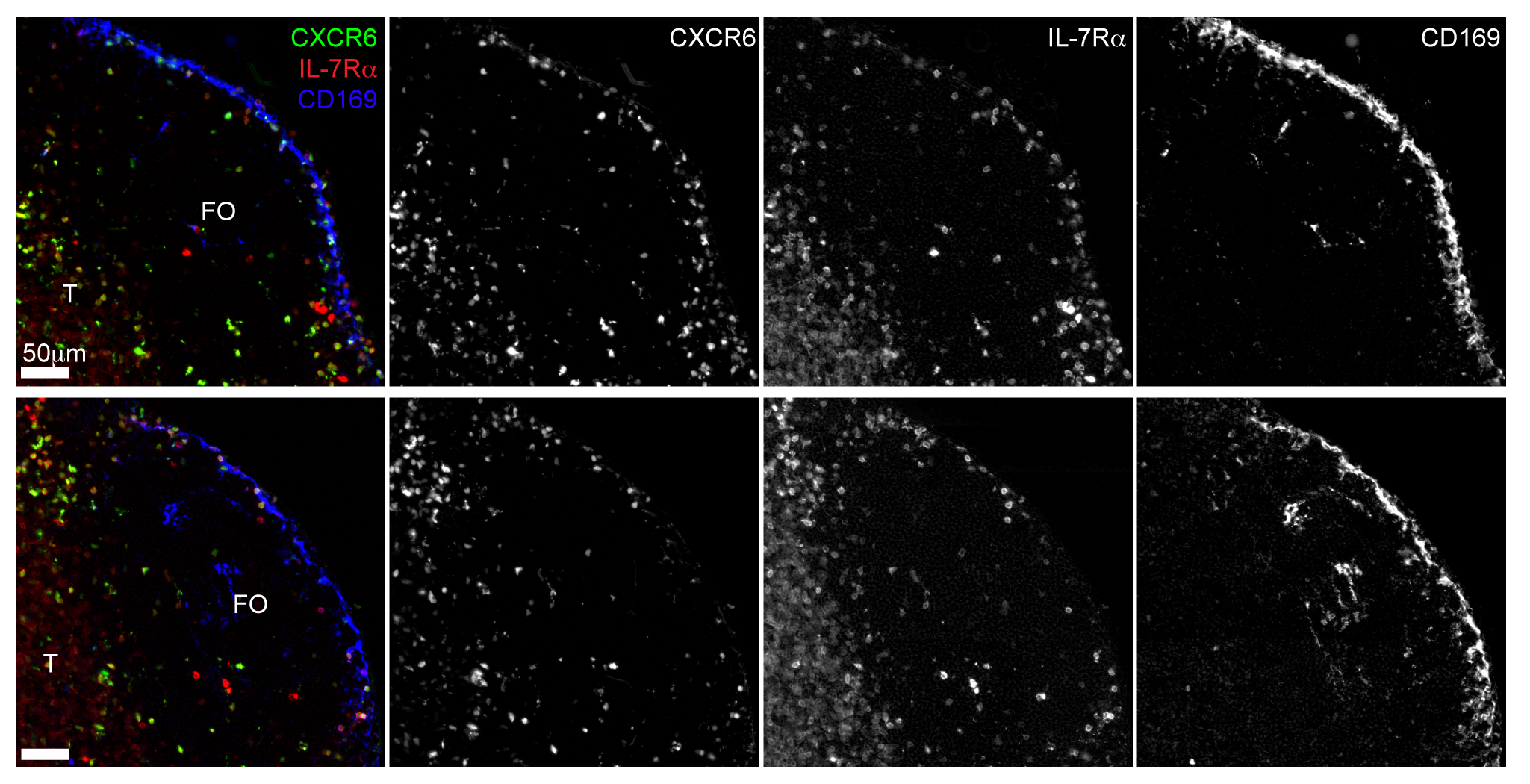

Supplement: Figure S3 — IL-7RαhiCXCR6hi lymphocytes at the subcapsular sinus and interfollicular regions. Immunofluorescence microscopy of lymph node sections from Cxcr6GFP/+ mice stained with anti-CD169 (blue) and anti-IL-7Rα (red) monoclonal antibodies. Two examples are shown and are representative of sections from four lymph nodes from two mice. FO, follicle; T, T zone. Scale bar = 50 µm. (TIF) [file pone.0038258.s003.tif]
